# Sertoli Cell-Specific Activation of Transforming Growth Factor Beta Receptor 1 Leads to Testicular Granulosa Cell Tumor Formation

**DOI:** 10.3390/cells12232717

**Published:** 2023-11-27

**Authors:** Xin Fang, Linfeng Nie, Satwikreddy Putluri, Nan Ni, Laurent Bartholin, Qinglei Li

**Affiliations:** 1 Department of Veterinary Integrative Biosciences, Texas A&M University, College Station, TX 77843, USA; 2 INSERM U1052, CNRS UMR5286, Centre de Recherche en Cancérologie de Lyon, Université Lyon 1, F-69000 Lyon, France; 3 Centre Léon Bérard, F-69008 Lyon, France

**Keywords:** transforming growth factor beta, Sertoli cell, testicular granulosa cell tumor, TGFBR1

## Abstract

The transforming growth factor β (TGFβ) superfamily, consisting of protein ligands, receptors, and intracellular SMAD transducers, regulates fundamental biological processes and cancer development. Our previous study has shown that sustained activation of TGFβ receptor 1 (TGFBR1) driven by anti-Mullerian hormone receptor type 2 (*Amhr2*)-Cre in the mouse testis induces the formation of testicular granulosa cell tumors (TGCTs). As *Amhr2*-Cre is expressed in both Sertoli cells and Leydig cells, it remains unclear whether the activation of TGFBR1 in Sertoli cells alone is sufficient to induce TGCT formation. Therefore, the objective of this study was to determine whether Sertoli cell-activation of TGFBR1 drives oncogenesis in the testis. Our hypothesis was that overactivation of TGFBR1 in Sertoli cells would promote their transdifferentiation into granulosa-like cells and the formation of TGCTs. To test this hypothesis, we generated mice harboring constitutive activation of TGFBR1 in Sertoli cells using anti-Mullerian hormone (*Amh*)-Cre. Disorganized seminiferous tubules and tumor nodules were found in *TGFBR1*^CA^; *Amh*-Cre mice. A histological analysis showed that Sertoli cell-specific activation of TGFBR1 led to the development of neoplasms resembling granulosa cell tumors, which derailed spermatogenesis. Moreover, TGCTs expressed granulosa cell markers including FOXL2, FOXO1, and INHA. Using a dual fluorescence reporter line, the membrane-targeted tdTomato (mT)/membrane-targeted EGFP (mG) mouse, we provided evidence that Sertoli cells transdifferentiated toward a granulosa cell fate during tumorigenesis. Thus, our findings indicate that Sertoli cell-specific activation of TGFBR1 leads to the formation of TGCTs, supporting a key contribution of Sertoli cell reprogramming to the development of this testicular malignancy in our model.

## 1. Introduction

Granulosa cell tumor (GCT) is a rare type of sex cord-stromal tumor that often occurs in the female gonad [1]. There are two histological subtypes of GCTs, the juvenile type and the adult type. GCTs may also develop in the testis [2]. Similar to ovarian GCTs, testicular GCTs (TGCTs) express granulosa cell markers including forkhead box L2 (FOXL2), forkhead box O1 (FOXO1), and inhibin alpha (INHA) [3]. The etiology of TGCTs is unclear; however, there is evidence supporting a potential link between juvenile TGCTs and chromosomal abnormalities and aberrant gonadal development [4,5]. Importantly, a point mutation of FOXL2 (402C > G; C134W) has been identified in adult ovarian GCTs as well as TGCTs, although the frequency of mutation appears lower in the latter [6,7]. In view of the rarity of these tumors, animal models are potentially important for dissecting tumorigenic events and helping understand the molecular basis of tumor initiation, development, and progression.

The transforming growth factor β (TGFβ) superfamily, consisting of multiple protein ligands, receptors, and intracellular SMAD transducers, regulates fundamental biological processes and cancer development [8,9]. TGFβ and BMP ligands bind to membrane-bound type II and type I receptors and induce signal transduction via the respective SMAD2/3 and SMAD1/5/8 in concert with a common SMAD, SMAD4. Dysregulation of TGFβ signaling provokes cancer development [2,10,11]. Studies using genetically modified mouse models demonstrate the critical role of TGFβ signaling in sex cord-stromal tumor development [2,12]. For example, conditional inactivation of SMAD1/5 or SMAD1/5/8 in ovarian granulosa cells leads to the formation of metastatic ovarian GCTs [13]. Direct evidence that links TGFβ signaling activation and GCT development stems from our previous work using mouse models expressing constitutively active TGFBR1 in their gonads driven by anti-Mullerian hormone receptor type 2 (*Amhr2*)-Cre (*TGFBR1^CA^*; *Amhr2-Cre)* [3,14]. Both females and males harboring *Amhr2*-Cre mediated overactivation of TGFBR1 develop gonadal tumors reminiscent of granulosa cell tumors revealed by both histological and molecular characterizations [3,14].

In *TGFBR1^CA^*; *Amhr2-Cre* males, abnormal Sertoli cell proliferation has been found during TGCT development [3]. Histological and molecular evidence suggests potential transdifferentiation from Sertoli cells to granulosa cells, in line with the notion that the two types of cells share the same progenitor cells [15]. Both Sertoli cells and Leydig cells play important roles in spermatogenesis [16,17]. Despite the different anatomic locations of Sertoli cells and Leydig cells (i.e., the tubular compartment versus the interstitial space), their interactions appear important for testicular functions [18]. As *Amhr2*-Cre is expressed in both Sertoli cells and Leydig cells [19], it remains unknown whether activation of TGFBR1 in Sertoli cells alone is sufficient to drive testicular GCT formation, despite the potent impact of TGFBR1 activation in these mice revealed by our previous study [3].

Thus, the objective of the current study was to determine whether Sertoli cell-specific activation of TGFBR1 promotes TGCT formation. To attain this aim, we generated mice with Sertoli cell-specific activation of TGFBR1 using *Amh*-Cre [20]. A dual fluorescence reporter mouse line was used to demonstrate the transdifferentiation of Sertoli cells into granulosa-like tumor cells. Our results show that Sertoli cell-specific activation of TGFBR1 results in the formation of testicular GCTs, supporting a key contribution of Sertoli cell reprogramming to the development of this testicular malignancy in the context of dysregulated TGFβ signaling.

## 2. Materials and Methods

### 2.1. Animals

Experiments using mice were approved by the Texas A&M University Institutional Animal Care and Use Committee. Mice containing *TGFBR1^CA^* were described previously [3,21]. The *TGFBR1^CA^* gene containing T204D, L193A, and P194A mutations was knocked into the X-linked hypoxanthine phosphoribosyltransferase 1 (*Hprt*) locus [21]. The expression of *TGFBR1^CA^* is driven by a CAG (human cytomegalovirus enhancer and chicken β-actin) promoter, and a LoxP-flanked stop sequence was engineered to allow for Cre-mediated expression of *TGFBR1^CA^* in a tissue/cell-specific manner. Detection of *TGFBR1^CA^* expression by Western blot was facilitated by using a hemagglutinin (HA) tag antibody to target the TGFBR1^CA^-HA fusion protein. *Amh*-Cre males were used to generate mice harboring Sertoli cell-specific expression of TGFBR1^CA^ (*TGFBR1^CA^*; *Amh-Cre*) (The Jackson Laboratory; stock no. 007915) [20]. The mTmG mice bearing a fluorescent reporter, tdTomato and EGFP, were purchased from the Jackson Laboratory (stock no. 007676) [22]. To generate *TGFBR1^CA^*; *Rosa^mTmG^*; *Amh-Cre* mice, *TGFBR1^CA^*; *Rosa^mTmG^* double homozygous females were produced and then bred with *Amh*-Cre males. Mice were maintained on C57BL/6; 129SvEv background and kept in the Comparative Medicine Program (CMP)’s Laboratory Animal Resources and Research Facility (LARR) at Texas A&M University. The facility was climate-controlled, and animals were under 12 h light:12 h dark cycles with free access to food and water.

### 2.2. Genotyping

The genotypes of *TGFBR1^CA^* mice were analyzed by genomic PCR, as described previously [21]. Gene-specific primers were used to detect *Amh*-Cre (5′-GCGGTCTGGCAGTAAAAACTATC-3′ and 5′-GTGAAACAGCATTGCTGTCACTT-3′; target band = ~100 bp) and mTmG (5′-CTTTAAGCCTGCCCAGAAGA-3′, 5′-TAGAGCTTGCGGAACCCTTC-3′, and 5′-AGGGAGCTGCAGTGGAGTAG-3′; wild-type band = 212 bp and mutant band = 128 bp) based on information provided by The Jackson Laboratory. Target amplicons were separated by electrophoresis and visualized using a VWR Gel Imager.

### 2.3. Tissue Collection and Processing

Testicular tissues were collected and fixed in 10% neutral-buffered formalin overnight at room temperature. Then, the fixed samples were washed with 70% ethanol before being processed and embedded in paraffin by the Histology Laboratory of Texas A&M School of Veterinary Medicine & Biomedical Sciences. The embedded tissues were sectioned at a thickness of 5 µm for hematoxylin and eosin (H.E) staining and immunohistochemical analyses.

### 2.4. Immunohistochemistry and Immunofluorescence

Immunohistochemistry was performed as described elsewhere [23]. Briefly, the paraffin sections were deparaffinized, rehydrated, and processed for heat-mediated antigen retrieval using sodium citrate buffer (pH = 6.0). Next, the sections were incubated with 0.3% H_2_O_2_ to inactivate the activity of endogenous peroxidase, blocked with non-immune serum, and incubated with primary antibodies overnight at 4 °C in a moisture chamber (Table 1). Incubation of secondary biotinylated antibody was performed at room temperature for 40 min. Signal amplification was achieved using the avidin–biotin complex (ABC) method based on the manufacturer’s instructions (Vector Laboratories, Burlingame, CA, USA). Vector NovaRED Substrate Kit was used to detect signals, followed by counterstaining with hematoxylin. Immunofluorescence microscopy was conducted as reported [23]. Upon completion of the staining, the sections were mounted using 4′,6-diamidino-2-phenylindole (DAPI)-containing ProLong Gold Slowfade media purchased from Thermo Scientific (Waltham, MA, USA). The sections were examined under a fluorescence microscope (IX73; Olympus, Waltham, MA, USA).

### 2.5. Western Blot

To prepare the testicular protein samples, testes from control and *TGFBR1^CA^*; *Amh-Cre* mice were homogenized using radioimmunoprecipitation assay buffer containing phosphatase and proteinase inhibitors [24]. The protein samples were quantified using bicinchoninic acid reagents (Thermo Scientific). Next, the proteins were separated using 12% Mini-PROTEAN TGX Precast Gels and processed for the Western blotting analysis detailed elsewhere [25]. After incubation with primary antibodies (Table 1), the blots were washed and further incubated with horseradish peroxidase (HRP)-conjugated anti-rabbit secondary antibody (1:20,000, Jackson ImmunoResearch, West Grove, PA, USA). Immobilon Western Chemiluminescent HRP Substrate (MilliporeSigma, Burlington, MA, USA) was used to develop signals, which were subsequently visualized using a Bio-Rad ChemiDoc MP Imaging System. To account for sample loading variations, the blots were stripped and reprobed with anti-β-actin (ACTB) antibody (Table 1). NIH ImageJ (1.53a) was used to quantify the target bands.

### 2.6. RNA Extraction and Quantitative Reverse Transcription PCR (qRT-PCR)

Total RNA samples were prepared from the testes of *TGFBR1^CA^*; *Amh-Cre* and control males using RNeasy Mini Kit (Qiagen, Germantown, MD, USA) based on the manufacturer’s manual. Reverse transcription was performed using Superscript III and qRT-PCR conducted using Bio-Rad iTaq Universal SYBR Green Supermix and primers for *Ctnnb1* (PrimerBank ID 6671684a1; 5′-ATGGAGCCGGACAGAAAAGC-3′ and 5′- CTTGCCACTCAGGGAAGGA-3′), SMAD family member 7 (*Smad7*) [14], connective tissue growth factor (*Ctgf*) [14], and serine (or cysteine) peptidase inhibitor, clade E, member 1 (*Serpine1*) [14], as described [23,26]. Relative quantification of gene expression was performed by normalizing the cycle threshold (CT) values of target genes against those of ribosomal protein L19 (*Rpl19*), and fold change was calculated [27]. Each sample was assayed in duplicate, with at least three biological replicates from each group.

### 2.7. Statistical Analysis

Comparisons of the means between two groups were made using an unpaired two-tailed *t*-test. Data are presented as mean ± SEM. Statistical significance was reported when the *p* value was <0.05. Significance levels were marked as * *p* < 0.05, ** *p* < 0.01, and *** *p* < 0.001. Ns indicates no significance detected.

## 3. Results

### 3.1. Generation of Mice with Sertoli Cell-Specific Activation of TGFBR1

**Figure 1:**
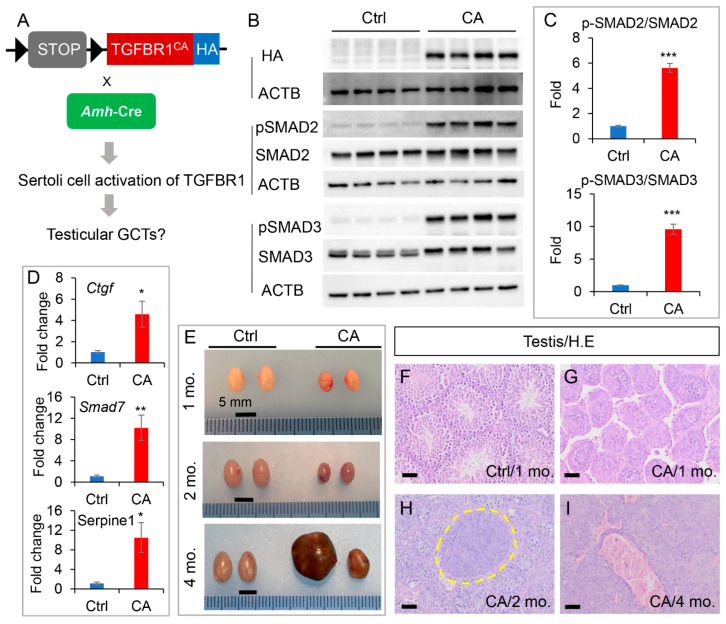
Development of testicular tumors induced by sustained activation of TGFBR1 in Sertoli cells. (**A**) A diagram depicting TGFBR1 overactivation using Cre-LoxP strategy. (**B**) Western blot analysis of *TGFBR1*^CA^ expression and SMAD2/3 activation in *TGFBR1^CA^*; *Amh-Cre* testes from 1-month-old mice. *n* = 4. ACTB served as an internal control. (**C**) Quantification of Western blot results. As HA was only expressed in *TGFBR1^CA^*; *Amh-Cre* testes but not in control groups, quantification of HA was not performed. *** *p* < 0.001. (**D**) Upregulation of TGFβ targets in *TGFBR1^CA^*; *Amh-Cre* testes from 1-month-old mice. The blue bars represent control mice, while the red bars show *TGFBR1^CA^*; *Amh-Cre* mice (**C**,**D**). Data are Mean ± SEM. *n* = 6. * *p* < 0.05 and ** *p* < 0.01. (**E**) Gross images of testes from 1, 2, and 4-month-old control and *TGFBR1^CA^*; *Amh-Cre* mice. Scale bar = 5 mm. (**F**–**I**) H.E. staining of control and *TGFBR1^CA^*; *Amh-Cre* testes at the age of 1, 2, and 4 months. Independent samples from at least three mice per genotype were examined. Ctrl, control. CA, *TGFBR1^CA^*; *Amh-Cre*. Scale bar = 50 µm (**F**–**I**).

To determine the impact of TGFBR1 activation in Sertoli cells, we generated mice with conditional expression of TGFBR1^CA^ using *Amh*-Cre [20] (Figure 1A). Expression of TGFBR1^CA^ in the testes of *TGFBR1^CA^*; *Amh-Cre* mice was confirmed by Western blot using HA tag antibody (Figure 1B). Consistent with TGFBR1 overactivation, expression levels of both phospho-SMAD2 and phospho-SMAD3 were increased in the testes of *TGFBR1^CA^*; *Amh-Cre* mice compared with the controls (Figure 1B,C). Total SMAD2, SMAD3, and ACTB were included as internal controls. To verify enhanced TGFβ signaling activity, we examined the expression of TGFβ downstream target genes including *Ctgf*, *Smad7*, and *Serpine1* using qRT-PCR. As expected, we found increased mRNA transcript levels of these genes in the testes of *TGFBR1^CA^*; *Amh-Cre* mice (Figure 1D).

### 3.2. Sertoli Cell-Specific Activation of TGFBR1 Leads to the Development of Testicular Tumors

As our previous studies show that conditional overexpression of TGFBR1^CA^ using *Amhr2*-Cre, which is expressed in both Sertoli cells and Leydig cells, promotes the formation of TGCTs [3], we sought to determine whether Sertoli cell-specific activation of TGFBR1 would lead to TGCT development in the current study. An analysis of gross anatomy showed that the testes of *TGFBR1^CA^*; *Amh-Cre* mice were smaller than those from the controls at both 1 and 2 months of age (Figure 1E). It was noteworthy that the testes of 2-month-old *TGFBR1^CA^*; *Amh-Cre* mice contained punctuated hemorrhagic lesions (Figure 1E). At 4 months of age, unilaterally enlarged testes were observed in *TGFBR1^CA^*; *Amh-Cre* mice, which developed hemorrhagic testicular tumors (Figure 1E). In contrast, all control mice were free of testicular tumors (Figure 1E).

Next, H.E. staining was performed to examine microscopic alterations of the testes. At 1 month of age, the size of seminiferous tubules was reduced in *TGFBR1^CA^*; *Amh-Cre* mice on cross sections of the testis compared with the controls (Figure 1F,G). Tumor foci containing neoplastic cells were evident in *TGFBR1^CA^*; *Amh-Cre* mice at 2 months of age (Figure 1H). At 4 months of age, highly disorganized seminiferous tubules and tumor nodules were observed in *TGFBR1^CA^*; *Amh-Cre* mice (Figure 1I). Histologically, the tumors had lobular structures resembling granulosa cell tumors observed in our previous studies [3] (Figure 1I).

**Figure 2:**
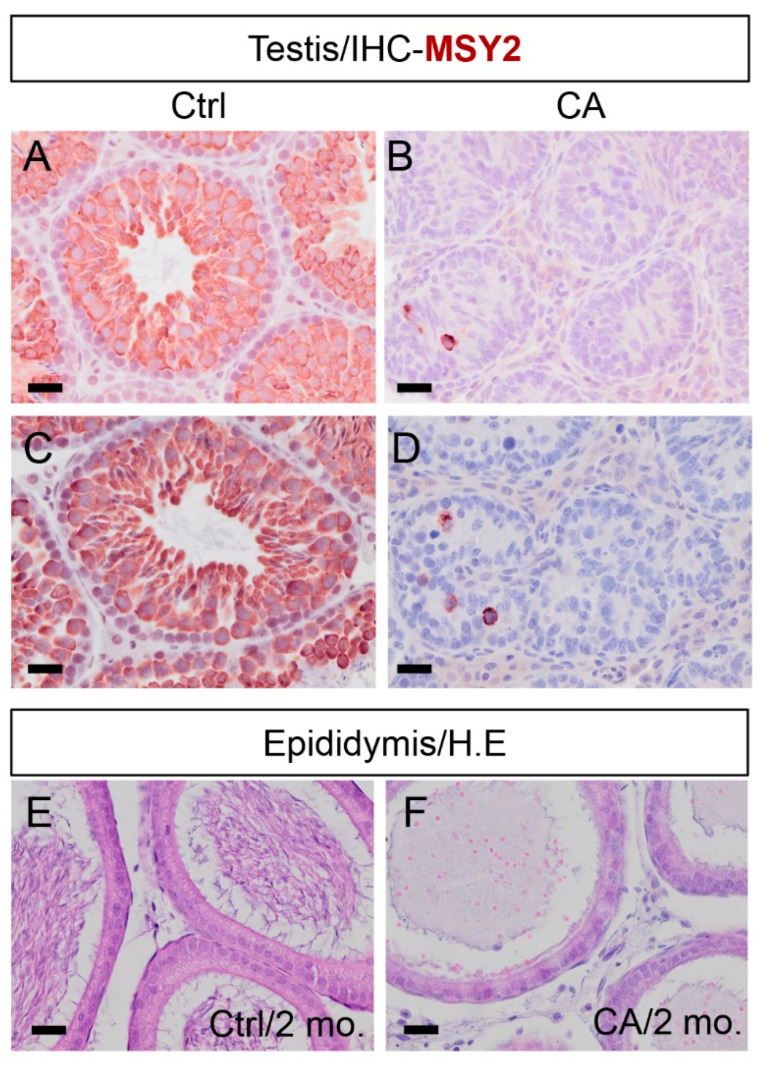
Loss of germ cells resulting from TGFBR1 overactivation in Sertoli cells. (**A**–**D**) Immunostaining of MSY2 in the testes of *TGFBR1*^CA^; *Amh*-Cre mice and controls. Testicular samples were analyzed using mice at the age of 1 (**A**,**B**) and 2 (**C**,**D**) months. (**E**,**F**) H.E. staining of epididymides from control and *TGFBR1*^CA^; *Amh*-Cre mice at the age of 2 months. Independent samples from at least three mice per genotype were examined. Scale bar = 25 µm (**A**–**F**).

To ascertain whether germ cell loss contributed to the reduced testicular size during early tumor development, we performed an immunohistochemical analysis of Y box protein 2 (MSY2), a DNA/RNA-binding protein that is abundantly expressed in the germ cell [28]. We found drastically reduced immunoreactive signals for MSY2 in the testes of *TGFBR1^CA^*; *Amh-Cre* mice at both 1 and 2 months of age (Figure 2B,D) compared with the controls (Figure 2A,C), indicating a loss of germ cells during testicular tumor development. The finding was reinforced by the absence of sperm in the epididymis of *TGFBR1^CA^*; *Amh-Cre* mice (Figure 2F). In contrast, the epididymis of control mice contained abundant sperm (Figure 2E). Therefore, tumor development in *TGFBR1^CA^*; *Amh-Cre* mice derails spermatogenesis.

### 3.3. Testicular Tumors Induced by TGFBR1 Overactivation in Sertoli Cells Resemble Granulosa Cell Tumors

**Figure 3:**
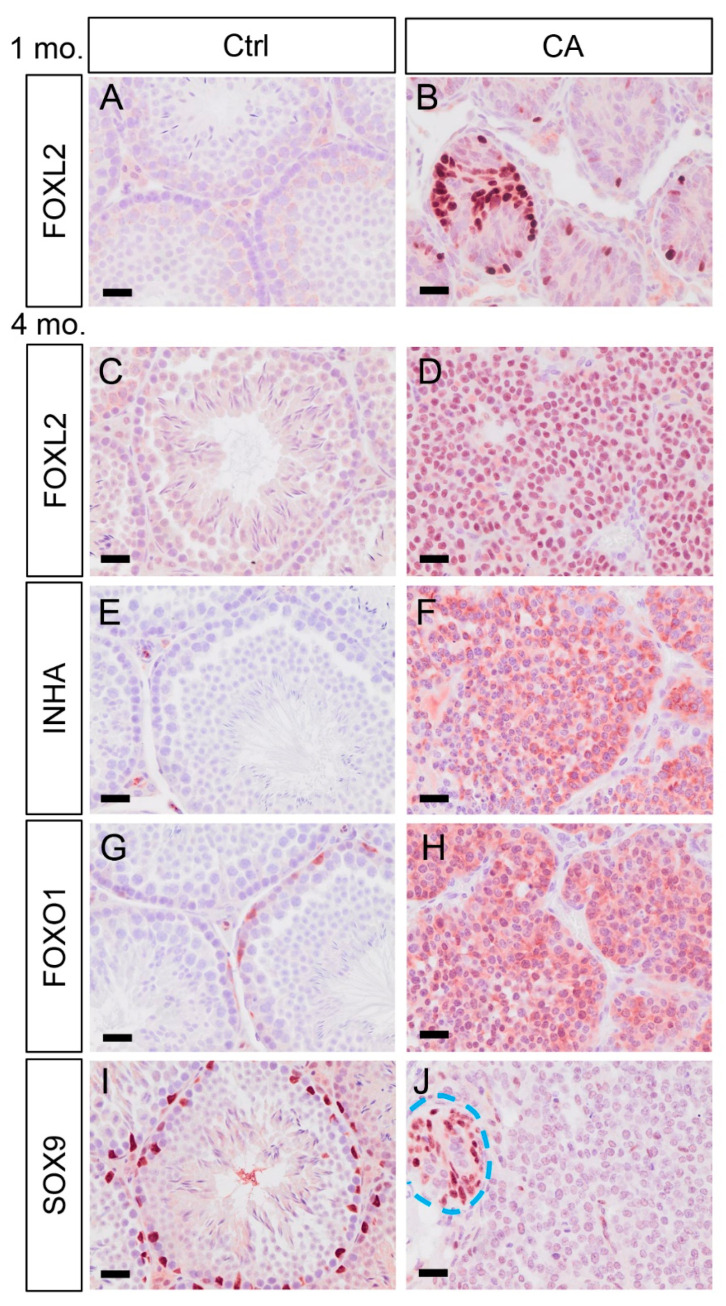
Testicular tumors induced by TGFBR1 overactivation in Sertoli cells express granulosa cell markers. (**A**,**B**) Immunolocalization of FOXL2 to the testes of control and *TGFBR1^CA^*; *Amh-Cre* mice at 1 month of age. (**C**–**J**) Immunolocalization of FOXL2, INHA, FOXO1, and SOX9 to the testes of control and *TGFBR1^CA^*; *Amh-Cre* mice at the age 4 months. Independent samples from at least three mice per genotype were examined. Scale bar = 25 µm (**A**–**J**).

**Figure 4:**
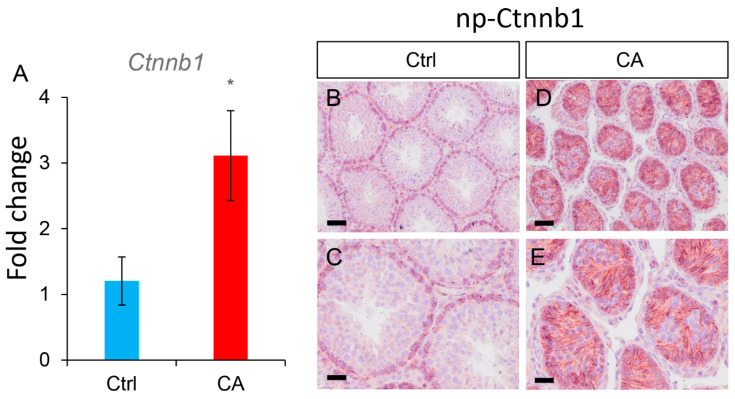
Increased expression of CTNNB1 in the testes of mice with Sertoli cell-specific activation of TGFBR1. (**A**) The transcript levels of *Ctnnb1* in the testes of 1-month-old control and *TGFBR1*^CA^; *Amh*-Cre mice. The blue bar represents control group (Ctrl), while the red bar indicates *TGFBR1^CA^*; *Amh-Cre* group (CA). Data are Mean ± SEM. *n* = 6. * *p* < 0.05. (**B**–**E**) Immunostaining of non-phospho CTNNB1 in the testes of control and *TGFBR1*^CA^; *Amh*-Cre mice at 1 month of age. (**C**,**E**) are higher power images showing some seminiferous tubules or abnormal tubule-like structures in (**B**,**D**), respectively. Independent samples from at least three mice per genotype were examined. Scale bar = 25 µm (**C**,**E**) and 50 µm (**B**,**D**).

To further determine the molecular identity of tumors observed in *TGFBR1^CA^*; *Amh-Cre* mice, we conducted immunostaining using antibodies against three granulosa cell tumor-expressed protein markers including FOXL2, INHA, and FOXO1 (Figure 3). Interestingly, the expression of FOXL2, a granulosa cell lineage marker, was only detectable within some abnormal seminiferous tubule-like structures in *TGFBR1^CA^*; *Amh-Cre* mice at 1 month of age (Figure 3B). At 4 months, testicular tumors were positively stained for all granulosa cell markers examined (Figure 3D,F,H). Testes from the control group containing normal seminiferous tubules were included (Figure 3A,C,E,G). In contrast to the expression of granulosa cell markers, SOX9 (a Sertoli cell marker) was not expressed in the parenchyma of the tumors, despite its apparent localization to the crowded nuclei within some dysplastic testicular tubules at 4 months of age (marked area with dotted line; Figure 3J). Spatially segregated Sertoli cell nuclei in the seminiferous tubules of controls were clearly labeled with anti-SOX9 antibody (Figure 3I). Moreover, the transcript expression of catenin (cadherin-associated protein) beta 1 (CTNNB1), the effector of WNT signaling critical for TGCT development [29], was increased in the testes of 1-month-old *TGFBR1^CA^*; *Amh-Cre* mice compared with controls (Figure 4A). Immunostaining of non-phospho (np)-CTNNB1 was strongly expressed in *TGFBR1^CA^*; *Amh-Cre* testes compared with the controls (Figure 4B–E). Together, our findings support that sustained activation of TGFBR1 in Sertoli cells induces the development of TGCTs.

### 3.4. TGFBR1 Overactivation in Sertoli Cells Promotes Sertoli Cell Proliferation and Transdifferentiation into Granulosa-like Cells

**Figure 5:**
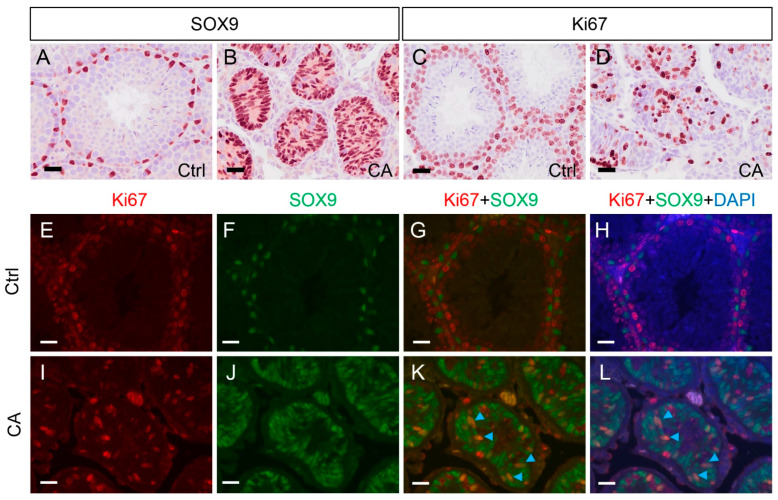
Increased Sertoli cell proliferation in testicular tumors of *TGFBR1^CA^*; *Amh-Cre* mice. (**A**–**D**) Immunostaining of SOX9 and Ki67 using testes from control and *TGFBR1^CA^*; *Amh-Cre* mice at 1 month of age. (**E**–**L**) Double immunofluorescence of Ki67 (red) and SOX9 (green) using testes from 1-month-old control and *TGFBR1^CA^*; *Amh-Cre* mice. Arrow heads in (**K**,**L**) show cells expressing both SOX9 and Ki67. DAPI staining (blue) shows the nuclei. Independent samples from at least three mice per genotype were examined. Scale bar = 25 µm (**A**–**D**) and 20 µm (**E**–**L**).

To determine the impact of TGFBR1 overactivation on cell identify, we performed immunostaining of SOX9 using testes from 1-month-old mice prior to gross tumor formation. Accompanied with the reduced size of the seminiferous tubule and the loss of germ cells in *TGFBR1^CA^*; *Amh-Cre* mice (Figure 2A,B), the nuclei of Sertoli cells were crowded and disoriented in the remnant seminiferous tubules (Figure 5B). The spatial distribution of Sertoli cells was unusual and in sharp contrast to that from age-matched controls (Figure 5A). To determine whether altered Sertoli cell distribution was accompanied by changes of cell proliferative properties, we examined the expression of Ki67, a cell proliferation marker. Ki67-positive cells were found within the seminiferous tubules of both control and *TGFBR1^CA^*; *Amh-Cre* mice (Figure 5C,D). However, expression of Ki67 in Sertoli cells was only detected in *TGFBR1^CA^*; *Amh-Cre* mice, but not in controls, using double immunofluorescence staining of Ki67 (red) and SOX9 (green) (Arrow heads; Figure 5E–L). Thus, proliferation of Sertoli cells upon overactivation of TGFBR1 contributes to testicular tumor development.

**Figure 6:**
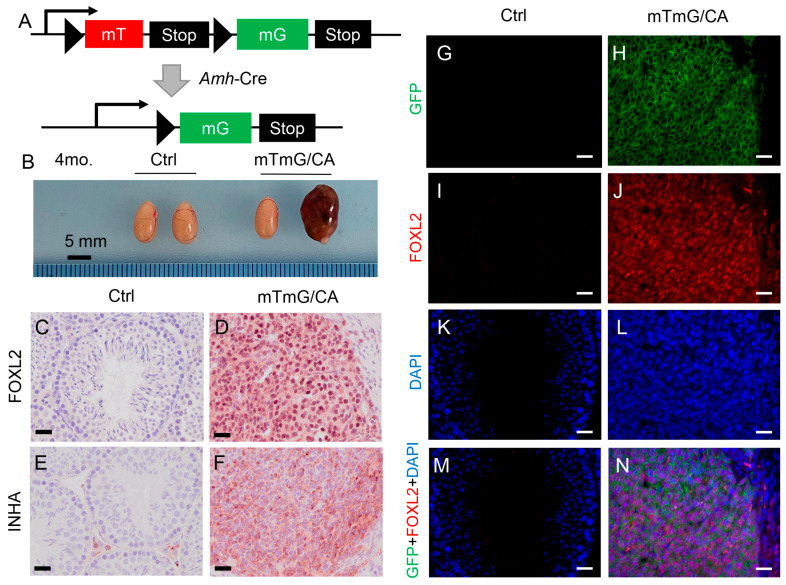
Transdifferentiation of Sertoli cells into granulosa-like tumor cells during tumorigenesis induced by TGFBR1 overactivation. (**A**) Genetic labeling of tumor cells using a double fluorescence reporter mouse line. A diagram depicts the generation of *TGFBR1^CA^*; *Rosa^mTmG^*; *Amh-Cre* mice. (**B**) Gross images of the testes from 4-month-old *TGFBR1^CA^*; *Rosa^mTmG^*; *Amh-Cre* mice and *TGFBR1^CA^*; *Rosa^mTmG^* controls. Scale bar = 5 mm. (**C**–**F**) Immunostaining of FOXL2 and INHA in the testes of *TGFBR1^CA^*; *Rosa^mTmG^*; *Amh-Cre* mice and controls at 4 months of age. Independent samples from at least three mice per genotype were examined. Scale bar = 25 µm (**C**–**F**). (**G**–**N**) Double immunofluorescence of GFP (green) and FOXL2 (red) using testes from 4-month-old control mice and *TGFBR1^CA^*; *Rosa^mTmG^*; *Amh-Cre* mice. DAPI staining (blue) shows the nuclei. The merged image in (**N**) shows the expression of FOXL2 in GFP-positive cells. Scale bar = 20 µm (**G**–**N**).

To further demonstrate that TGFBR1 overactivation promotes Sertoli cell transdifferentiation into granulosa-like tumor cells, we took advantage of the membrane-targeted tdTomato (mT)/membrane-targeted EGFP (mG) mouse, a dual fluorescence reporter. In this reporter line, Cre-negative cells express tdTomato, a red fluorescent protein, while Cre-positive Sertoli cells express GFP (Figure 6A). First, we generated *TGFBR1^CA^*; *Rosa^mTmG^*; *Amh-Cre* mice and controls (*TGFBR1^CA^*; *Rosa^mTmG^*) (Figure 6A). The expression of GFP in Sertoli cells of *TGFBR1^CA^*; *Rosa^mTmG^*; *Amh-Cre* mice was confirmed using immunostaining (PD14; Supplementary Figure S1A,B). As expected, testicular tumors were developed in *TGFBR1^CA^*; *Rosa^mTmG^*; *Amh-Cre* mice when examined at 4 months of age (Figure 6B), similar to *TGFBR1^CA^*; *Amh-Cre* mice. Tumor nodules in *TGFBR1^CA^*; *Rosa^mTmG^*; *Amh-Cre* mice were also positively stained for FOXL2 and INHA (Figure 6C–F), consistent with the development of TGCTs. Next, double immunofluorescence was conducted using antibodies against GFP labeling Sertoli cells and FOXL2 labeling granulosa cells. As expected, expressions of GFP and FOXL2 were not detectable in control testes (Figure 6G,I,K,M). In contrast, co-localization of GFP and FOXL2 was found in the testes of *TGFBR1^CA^*; *Rosa^mTmG^*; *Amh-Cre* mice (Figure 6H,J,L,N). An immunohistochemical analysis was also performed using serial sections to show that the tumor in *TGFBR1^CA^*; *Rosa^mTmG^*; *Amh-Cre* mice expressed GFP (Supplementary Figure S1C–H). Collectively, we herein provide genetic evidence supporting the transdifferentiation of Sertoli cells into granulosa-like tumor cells during tumorigenesis induced by TGFBR1 overactivation in these cells (Supplementary Figure S2).

## 4. Discussion

We previously reported that overactivation of TGFβ signaling using *Amhr2*-Cre expressed in both Sertoli cells and Leydig cells promotes the development of TGCTs, revealing the oncogenic role of dysregulated TGFβ signaling in the testis [3]. The development of TGCTs in *TGFBR1^CA^*; *Amhr2-Cre* mice is intriguing because of the absence of the granulosa cell type and the expression of its lineage marker FOXL2 in normal testis. To further understand TGCT development in the context of enhanced TGFβ signaling, we created an additional mouse model with Sertoli cell-specific activation of TGFBR1. Our results showed that the expression of constitutively active TGFBR1 in Sertoli cells using *Amh*-Cre also led to the development of TGCTs. By using a dual fluorescence reporter mouse line, we provided further evidence supporting that Sertoli cells transdifferentiated toward a granulosa cell fate during tumorigenesis. These findings indicate the significant impact of TGFβ signaling activation in Sertoli cells on the development of TGCTs.

Sertoli cells are nursing cells in the testis, and the number of them is important for testicular size and spermatogenesis [16,30]. It has been shown that ablation of Sertoli cells in the testis causes a loss of germ cells [31]. Interestingly, increased Sertoli cell proliferation induced by constitutively active TGFBR1 also led to a loss of germ cell populations, as observed in our previous report [3] and the current study. Detailed mechanisms underlying germ cell loss are unclear. However, it is conceivable that impaired proliferation/differentiation of Sertoli cells compromised their functions, thereby affecting Sertoli cell-germ cell communications and germ cell maintenance. Granulosa cells are the ovarian counterpart of Sertoli cells and play a key role in oocyte development [32]. Sertoli cells and granulosa cells appear to arise from common precursor cells during gonadal development [15,33]. One intriguing phenomenon observed in sex cord-stromal tumors is the altered identify of granulosa cells or Sertoli cells, suggesting potential transdifferentiation between the two cell types. For instance, SOX9-positive cells are present in ovarian GCTs resulting from depletion of FOXO1/3 or overactivation of TGFβ signaling [14,34]. On the other hand, constitutive activation of TGFBR1 in the testis using *Amhr2*-Cre recombinase promotes the formation of TGCTs that express markers of granulosa cells [3]. A question raised by our previous study is whether constitutive activation of TGFBR1 in Sertoli cells is sufficient to drive tumorigenesis in the testis.

Our histological and immunohistochemical analyses showed that overactivation of TGFBR1 in Sertoli cells led to the formation of sex cord-stromal tumors similar to those observed in *TGFBR1^CA^*; *Amhr2-Cre* mice [3]. Both *TGFBR1^CA^*; *Amh-Cre* and *TGFBR1^CA^*; *Amhr2-Cre* male mice showed proliferation of otherwise mitotically inactive Sertoli cells, impaired germ cell development, increased expression of WNT signaling effector, and expression of granulosa cell lineage marker FOXL2 in the testes [3]. To genetically label Sertoli cells and determine their potential contribution to TGCT development, we took advantage of a dual fluorescence reporter mouse line and generated *TGFBR1^CA^*; *Rosa^mTmG^*; *Amh-Cre* mice. The results showed that some tumor nodules expressed GFP, suggesting the transdifferentiation of Sertoli cells into granulosa-like tumor cells. Our findings highlight the key contribution of Sertoli cells to the development of TGCTs upon overactivation of TGFβ signaling.

A landmark study has discovered that nearly all human adult ovarian GCTs bear a somatic missense mutation of *FOXL2* (c.402C > G; p.C134W) [6]. In vitro studies suggest that the pathognomonic *FOXL2* mutation (C134W) causes altered expression of genes encoding TGFβ signaling components [12,35,36]. The mutant FOXL2 interacts with TGFβ-related SMADs in human granulosa cell line or GCT cell lines [37,38]. The in vivo consequence of *FOXL2* mutation in ovarian GCT development has been demonstrated using a mouse model, where ovarian GCTs form in mice harboring a *Foxl2* mutation (C130) corresponding to the orthologous transcript that encodes the human C134 [39]. These studies suggest that FOXL2^C134W^ acts as a gain-of-function mutation inducing an oncogenic effect in ovarian granulosa cells [39]. More evidence appears to support this concept [40], although a recent study points to a tumor suppressive role of FOXL2^C134W^ in ovarian granulosa cells [41]. Recent findings indicate that *FOXL2* mutation may cause altered TGFβ/activin signaling, increased secretion of estradiol, and altered cell properties that promote tumorigenesis [12,35,36,37,42,43,44]. However, how *Foxl2* mutation links to the dysregulation of cell signaling cascades during granulosa cell tumorigenesis is elusive. Consistent with the role of TGFβ signaling in GCTs, a recent study shows that the mutant FOXL2^C134W^ is capable of binding SMAD4, leading to the formation of a protein complex containing FOXL2^C134W^, SMAD2/3, and SMAD4 [38]. This complex can induce the transcription of genes associated with epithelial-to-mesenchymal transition (EMT) via binding a novel DNA motif [38]. That the action of mutant FOXL2^C134W^ depends on TGFβ-SMAD signaling reinforces a critical role of TGFβ signaling in ovarian GCT development [38].

Despite the reported detection of FOXL2 mutation in an adult TGCT patient, a recent study has revealed that adult TGCTs rarely contain FOXL2 mutation and therefore are molecularly heterogeneous [45]. Unlike the *Foxl2* mutant female mice, male mice with *Foxl2* mutation show normal testis development and fertility and are free of gonadal tumors [39], supporting the notion that the genetic causes may be different between ovarian GCTs and testicular GCTs. The current study, together with our previous reports, revealed that overactivation of TGFBR1 in granulosa cells or Sertoli cells inevitably leads to GCT development, highlighting a common mechanism of dysregulated TGFβ signaling in the development of these tumors. TGFβ signaling plays a key role in EMT during tumorigenesis [46]. Of note, both *Ctgf* and *Serpine1* are involved in EMT [47,48] and were upregulated in the testes of *TGFBR1^CA^*; *Amh-Cre* mice. Thus, future studies are necessary to determine whether EMT contributes to the development of TGCTs in our model. The strengths of the present study include the use of Sertoli cell-specific Cre recombinase, overactivation of TGFBR1 using a well-established in vivo model system, and genetic labeling of Sertoli cells with a dual fluorescence reporter line. However, a limitation of our model is that dysregulation of TGFβ signaling in granulosa cell tumors does not necessarily result from overactivation of TGFBR1. Nevertheless, our model with cell type-specific activation of TGFBR1 provided compelling evidence that TGFβ signaling-associated Sertoli cell reprogramming might be a key event in the oncogenesis of TGCTs.

Because ovarian GCTs and TGCTs share some features in histopathology and dysregulated signaling pathways, we have postulated that comparative analyses may represent an useful approach to study these rare tumors [2]. Of note, a recent clinical phase I study has evaluated the effect of activin A inhibitor in human adult GCT patients [49]. More recently, new evidence indicates that inhibition of TGFβ signaling using an orally available small molecule inhibitor for TGFBR1, TP-6379, in KGN cells harboring FOXL2 mutation effectively reduces cell growth [50]. Thus, targeting TGFβ signaling may represent a novel approach to treat granulosa cell tumors. Significant progress has been made in developing effective targeting options for the TGFβ signaling pathway. So far, various pharmacological strategies have been used for manipulating cancer-related TGFβ signaling, including, but not limited to, small molecule inhibitors that suppress the receptor kinase activity, antibodies that block ligand-receptor binding, and antisense oligonucleotides that target TGFβ signaling elements [51]. Based on our findings, it will be interesting to explore the therapeutic effect of targeting TGFβ signaling on ovarian GCT development in both *Foxl2* mutant mice and TGFBR1 constitutively active females and TGCT development in our current mouse model. Further comparative studies may shed new light on the treatment of GCTs in both sexes through targeting a central pathway involved in the pathogenesis of sex cord-stromal tumors. Importantly, mechanisms that contribute to the antitumoral effect and/or the side effect of a compound can be further evaluated in these animal models. The development of novel targeted therapies may also allow for more effective management of the risk of GCT recurrence.

## Figures and Tables

**Table 1:** Primary antibody information.

Name	Company	Cat.#	Species	IHC/IF	WB
ACTB	Cell Signaling (Danvers, MA, USA)	12262	Mouse		1:1000
FOXO1	Cell Signaling	2880	Rabbit	1:800	
FOXL2	Abcam (Cambridge, UK)	Ab5096	Goat	1:1500	
FOXL2	Abcam	Ab246511	Rabbit	1:500	
GFP	Novus Biologicals (Littleton, CO, USA)	NB600-308SS	Rabbit	1:1000	
GFP	Cell Signaling	2956S	Rabbit	1:50	
HA	Roche (Basel, Switzerland)	12013819001	Rat		1:500
INHA	Biorad (Hercules, CA, USA)	MCA951ST	Mouse	1:300	
MSY2	Abcam	Ab33164	Rabbit	1:500	
Non-phospho CTNNB1	Cell Signaling	19807	1:1500		
Phospho-SMAD2	Cell Signaling	3101	Rabbit		1:1000
Phospho-SMAD3	Abcam	Ab52903	Rabbit		1:2000
SMAD2	Cell Signaling	5339	Rabbit		1:1000
SMAD3	Abcam	Ab28379	Rabbit		1:1000
SOX9	Millipore (Burlington, MA, USA)	Ab5535	Rabbit	1:2000	

## Data Availability

Data are included in the article and Supplementary Materials.

## Supplementary Materials

The following supporting information can be downloaded at: https://www.mdpi.com/article/10.3390/cells12232717/s1, Figure S1: GFP expression in the testes of *TGFBR1*^CA^; *Rosa*^mTmG^; *Amh*-Cre mice. Figure S2: A working model depicting TGFBR1 overactivation in Sertoli cells and TGCT development.
