# Supplementary material for: Sertoli Cell-Specific Activation of Transforming Growth Factor Beta Receptor 1 Leads to Testicular Granulosa Cell Tumor Formation
Source: Cells. 2023 Nov 27;12(23):2717. doi: 10.3390/cells12232717 (PMC10706251; doi:10.3390/cells12232717)
Supplement: Supplementary file 1 [file cells-12-02717-s001.zip › cells-2705958-supplementary.pdf]

Sertoli Cell-Specific Activation of Transforming Growth Factor Beta Receptor 1 Leads to Testicular Granulosa Cell Tumor Formation

Xin Fang[1], Linfeng Nie[1], Satwikreddy Putluri[1], Nan Ni[1], Laurent Bartholin[2,3], Qinglei Li[1,*]

Supplementary Materials

Figure S1. GFP expression in the testes of *TGFBR1*^{CA}; *Rosa*^{mTmG}; *Amh-Cre* mice.

Figure S2. A working model depicting TGFBR1 overactivation in Sertoli cells and TGCT development.

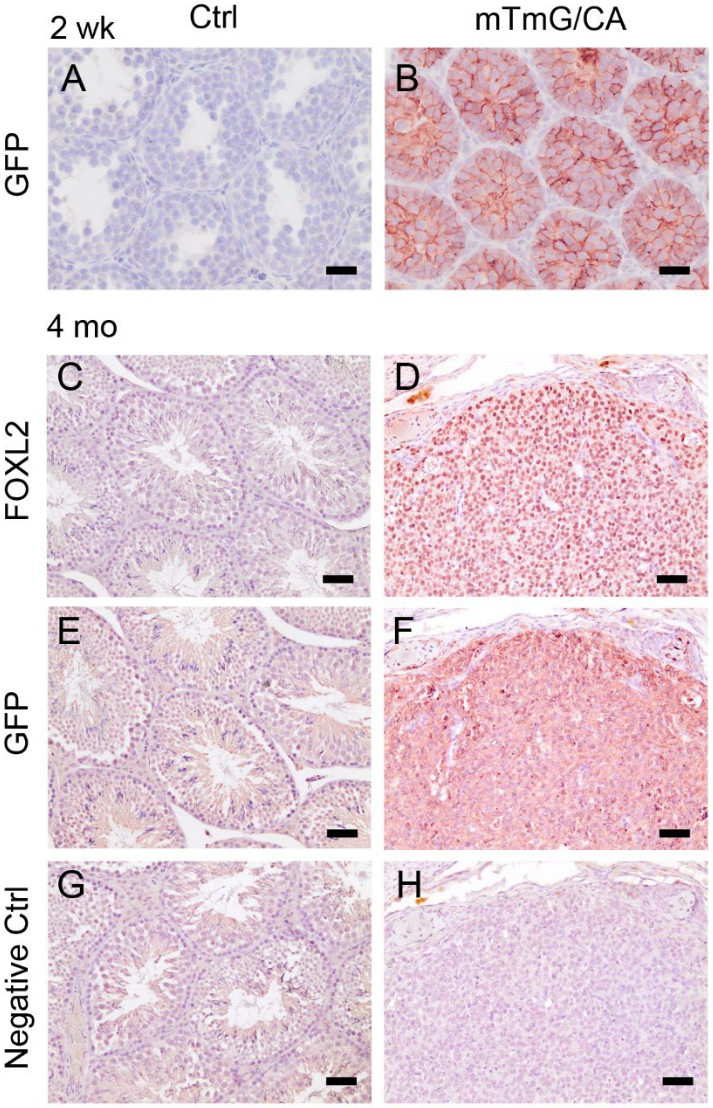

Figure S1. GFP expression in the testes of *TGFBR1^{CA}*; *Rosa^{mTmG}*; *Amh-Cre* mice. (**A, B**) Immunostaining of GFP in the testes of control and *TGFBR1^{CA}*; *Rosa^{mTmG}*; *Amh-Cre* mice at 2 weeks of age. (**C-H**) Immunostaining of FOXL2 and GFP in the testes of control and *TGFBR1^{CA}*; *Rosa^{mTmG}*; *Amh-Cre* mice at 4 months of age. Scale bar = 25 μm (A, B) and 50 μm (C-H).

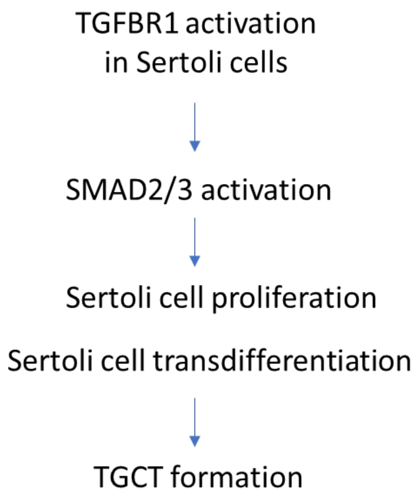

Figure S2. A working model depicting TGFBR1 overactivation in Sertoli cells and TGCT development.
